# *Xanthomonas citri* MinC Oscillates from Pole to Pole to Ensure Proper Cell Division and Shape

**DOI:** 10.3389/fmicb.2017.01352

**Published:** 2017-07-19

**Authors:** André S. G. Lorenzoni, Giordanni C. Dantas, Tessa Bergsma, Henrique Ferreira, Dirk-Jan Scheffers

**Affiliations:** ^1^Department of Molecular Microbiology, Groningen Biomolecular Sciences and Biotechnology Institute, University of Groningen Groningen, Netherlands; ^2^Departamento de Bioquímica e Microbiologia, Instituto de Biociências, Universidade Estadual Paulista Rio Claro, Brazil

**Keywords:** *Xanthomonas citri*, MinC, FtsZ, ZapA, ParB, peptidoglycan, citrus canker

## Abstract

*Xanthomonas citri* (Xac) is the causal agent of citrus canker, a disease that affects citrus crops and causes economic impact worldwide. To further characterize cell division in this plant pathogen, we investigated the role of the protein MinC in cell division, chromosome segregation, and peptidoglycan incorporation by deleting the gene *minC* using allele exchange. Xac with *minC* deleted exhibited the classic Δ*min* phenotype observed in other bacteria deleted for *min* components: minicells and short filamentation. In addition we noticed the formation of branches, which is similar to what was previously described for *Escherichia coli* deleted for either *min* or for several low molecular weight penicillin-binding proteins (PBPs). The branching phenotype was medium dependent and probably linked to gluconeogenic growth. We complemented the *minC* gene by integrating *gfp-minC* into the *amy* locus. Xac complemented strains displayed a wild-type phenotype. In addition, GFP-MinC oscillated from pole to pole, similar to MinCD oscillations observed in *E. coli* and more recently in *Synechococcus elongatus*. Further investigation of the branching phenotype revealed that in branching cells nucleoid organization, divisome formation and peptidoglycan incorporation were disrupted.

## Introduction

*Xanthomonas citri* subsp. *citri* (Xac) is the causal agent of citrus canker, a severe disease that affects citrus crops, decreases fruit production and causes economic losses (Gottwald et al., 2002). This disease is currently present in South and North America, Asia, Africa, and Oceania (Stover et al., 2014; Davis et al., 2015; Leduc et al., 2015; Behlau et al., 2016). The current strategy to combat citrus canker in the state of São Paulo, Brazil, the largest producer of concentrate orange juice in the world, comprises the eradication of symptomatic trees along with spraying copper-containing bactericides in a radius of 30 m having the symptomatic tree as the center point (Gottwald et al., 2002; Behlau et al., 2011). However, this strategy is costly and has limited effectiveness (Behlau et al., 2012, 2016).

The genome of Xac was fully sequenced 14 years ago, opening up several possibilities for molecular and genetic characterization of this plant pathogen (da Silva et al., 2002). Since then, some studies have expanded upon the knowledge we have about biological processes in Xac, mostly concerning pathogenicity mechanisms (Alegria et al., 2004; Casabuono et al., 2011; Li and Wang, 2012; Huang et al., 2013; Alexandrino et al., 2016). Recently, new tools for protein expression in Xac have been developed (Martins et al., 2010; Ucci et al., 2014; Lacerda et al., 2017), enabling studies on chromosome segregation and cell division. These cellular systems represent potential targets for antimicrobials, as the proteins involved in such processes share little homology with eukaryotic equivalents (Pan et al., 2006; Vollmer, 2006; Sass and Brötz-Oesterhelt, 2013; Broughton et al., 2016). Anti-Xac compounds that disrupt the localization of the cell division proteins ZapA and FtsZ *in vivo*, and that act on FtsZ *in vitro*, also affect localization of ParB (Silva et al., 2013; Król et al., 2015). Xac cells expressing truncated forms of ParB exhibit a filamentation phenotype (Ucci et al., 2014). Inhibition of cell division in other bacteria like *E. coli* and *B. subtilis* leads to filamentation without chromosome segregation defects (Margolin, 2005), suggesting a difference in the relation between cell division and chromosome segregation in Xac.

FtsZ is the central protein of the cell division machinery (Margolin, 2005). At the start of cell division, FtsZ assembles a ring like structure at midcell called the Z ring. Z ring formation is followed by mid-cell constriction that generates two daughter cells. FtsZ is conserved among prokaryotic cells, yet different mechanisms have evolved in different bacteria that ensure that the Z ring assembles at midcell, at a specific time during the cell cycle, and does not constrict over the nucleoids. In the most studied gammaproteobacterium, *Escherichia coli*, Z ring localization is mediated by the Min system that consists of the proteins MinC, MinD, and MinE (Shih and Zheng, 2013). MinC forms a complex with MinD to inhibit FtsZ function at the cell poles (Ghosal et al., 2014). The localization of MinC is mediated by MinD, which forms a membrane-associated complex at one of the cell poles. MinE assembles at midcell in a ring like structure, and then cycles back and forth toward the cell poles stimulating the dissociation of the MinC/MinD complex. As soon as MinC/D dissociate from one of the poles, they are re-oriented to the opposite cellular pole in such a way that MinC, the FtsZ inhibitor, resides closer to the cellular poles far longer than it is in transit between them. This behavior creates a concentration gradient through the cell with MinCD lowest at midcell, which enables Z ring formation at this site (Rowlett and Margolin, 2013). For a long time, MinCD oscillation had only been identified in *E. coli*, but recently it has also been observed in *Synechococcus elongatus* (MacCready et al., 2016), as well as MinD oscillation in *Vibrio cholerae* (Galli et al., 2016). Although inhibition of polar Z ring formation by MinCD is conserved in many bacteria, oscillation of MinCD seems restricted to bacteria that also contain MinE. In bacteria that do not have MinE, MinCD is anchored to the poles via other proteins such as DivIVA/MinJ (Bramkamp and van Baarle, 2009).

Another mode of control of Z ring formation is the partially redundant nucleoid occlusion system, in *E. coli* mediated by SlmA (Bernhardt and de Boer, 2005). SlmA binds to specific DNA sites and depolymerizes FtsZ, and this way the Z ring cannot assemble over a nucleoid (Cabré et al., 2015). In several alphaproteobacteria including the model organism *Caulobacter crescentus*, the DNA-binding protein ParB colocalizes with the edges of the nucleoids to drive chromosome segregation (Mohl and Gober, 1997; Ucci et al., 2014). Then ParB forms a complex with MipZ, a FtsZ inhibitor that tracks along the ParB-chromosomal origin region (the bacterial centromere) in such a way that the inhibitory effect of MipZ will be concentrated at the cell poles and distal from the mid-cell where the divisional septum will be assembled (Thanbichler and Shapiro, 2006).

Although Xac is a member of the gammaproteobacteria, in terms of chromosome segregation, it seems, at least mechanistically, related to *C. crescentus* and *V. cholerae*, as in these bacteria chromosome segregation is asymmetric. However, Xac does not have an obvious MipZ homolog. In terms of FtsZ regulation, Xac is closer to *E. coli* and *V. cholerae*, due to the presence of the Min system composed of the MinCDE proteins (Galli et al., 2016), although Xac doesn't have an obvious SlmA homolog that could mediate nucleoid occlusion (Ucci et al., 2014).

In this study, we have made a Xac mutant deleted for *minC* and observed that this mutant not only forms minicells but also exhibits branching. This phenotype has also been described in *E. coli* deleted for *min* (Akerlund et al., 1993; Gullbrand et al., 1999). In *E. coli*, branching is dependent on the medium composition, and seems associated (or more prominent) with minimal media and the presence of casaminoacids, and a disturbed localization of the nucleoids (Akerlund et al., 1993). Branching increased in cells grown in the presence of low concentrations of beta-lactam antibiotics, indicating that interfering with PBP function increases this phenotype (Gullbrand et al., 1999). This was confirmed in a series of papers from the Young laboratory, who observed a similar branching phenotype in *E. coli* deleted for several low-molecular-weight penicillin-binding proteins (LMW PBPs), most notably PBP5, the major DD-carboxypeptidase (Nelson and Young, 2000, 2001). Branching most likely results from aberrant positioning of FtsZ and concomitant synthesis of so-called “inert peptidoglycan” (iPG), which is not associated with cell division. iPG is consistently observed at the tips of branches and at branch sites (Varma and Young, 2004, 2009; Varma et al., 2007; Potluri et al., 2012).

In order to study cell division regulation in Xac in more detail, we further analyzed our *minC* mutant. In this paper we describe the relation between cell division and the regulatory Min system, which is critical for preventing polar division by regulating the position of the FtsZ ring.

## Methods

### Bacterial strains and growth conditions

Bacterial strains and plasmids are listed in Table 1. Xac was cultivated at 30°C in various media: nutrient yeast glycerol broth (NYGB, peptone 5 g/L; yeast extract 3 g/L; glycerol 20 g/L) or on plates containing agar (15 g/L, NYGA) supplemented with D-glucose (1% w/v) or L-arabinose (0.05% w/v) when required; nutrient yeast citrate broth (NYCB, peptone 5 g/L; yeast extract 3 g/L; glycerol 2.46 g/L; trisodium citrate 0.44 g/L); Xam1 medium (per liter: 2.46 g glycerol, 0.247 g MgSO_4_·7H_2_O, 1.0 g (NH_4_)_2_SO_4_, 4.5 g KH_2_PO_4_, 10.5 g K_2_HPO_4_, 0.5 g Na_3_C_6_H_5_O_7_·2H_2_O, 0.3 g casaminoacids, 1 g BSA, pH 5.4 with HCl); or Xamg1 medium which is identical to Xam1 except for glycerol which is at 20 g/L. LB 0% is Lysogeny Broth without NaCl (tryptone 10 g/L; yeast extract 5 g/L), whereas LB 0.5% contains NaCl 5 g/L.

**Table 1:** List of strains and plasmids.

**Strains**	**Relevant characteristics**	**References**
*X. citri* subsp. *citri* (Xac)	Wild type strain 306 (Xac); Ap^R^	IBSBF-1594^*^ da Silva et al., 2002; Schaad et al., 2005, 2006
*E. coli* DH10B	Cloning strain	Invitrogen
Xac Δ*minC*	Xac Δ*minC*; Ap^R^	This work
Xac *amy::gfp*	Xac with pGCD21 integrated in *amy*; Ap^R^ Km^R^	This work
Xac Δ*minC amy::gfp*	Xac Δ*minC* with pGCD21 integrated in *amy*; Ap^R^ Km^R^	This work
Xac Δ*minC amy::gfp-minC*	Xac Δ*minC* with pGCD2c integrated in *amy*; Ap^R^ Km^R^	This work
Xac *amy::gfp-zapA*	Xac with pPM2a-ZapA integrated in *amy*; Ap^R^ Km^R^ (former name: pPM2a-XAC3408)	Martins et al., 2010
Xac Δ*minC amy::gfp-zapA*	Xac Δ*minC* with pPM2a-ZapA integrated in *amy*; Ap^R^ Km^R^	This work
Xac *parB::parB-gfp*	Xac with pPM7g-parB integrated in *parB*; Ap^R^ Km^R^ (former name: *parB*::pAPU3)	Ucci et al., 2014
Xac Δ*minC parB::parB-gfp*	Xac Δ*minC* with pPM7g-parB integrated in *parB*; Ap^R^ Km^R^	This work
**PLASMIDS**		
pPM2a and pPM7g	GFP expression vectors; *xylR pxyl gfpmut1* Ap^R^ Km^R^	Martins et al., 2010
pAPU3	pPM7g-*parB*: *xylR pxyl parB-gfpmut1* Ap^R^ Km^R^	Ucci et al., 2014
pHF5Ca	TAP-tag expression vector; *xylR pxyl tap1479* Ap^R^ Km^R^	Ucci et al., 2014
pEB304	pBAD derivative and source of the *araC*-p*ara-acp-tap1479* cassette; Ap^R^	Gully et al., 2003
pNPTS138	*Bacillus subtilis sacB* gene; Km^R^; suicide vector in Xac	Prof. L. Shapiro (Stanford University, USA)
pGCD21	Derivative of pHF5Ca; *araC*-p*ara-gfpmut1*; *amy106-912*; Ap^R^ Km^R^; integrative vector in Xac	This work GenBank KU678206
pLAL1	*araC*-p*ara-acp-tap1479*; Gm^R^; replicative vector in Xac	GenBank KP696472 Lacerda et al., 2017
pGCD1C	Derivative of pLAL1; *araC*-p*ara-minC*; Gm^R^; replicative vector in Xac	This work
pGCD19	GFP expression vector; *xylR pxyl gfpmut1* Ap^R^ Km^R^	This work
pGCD2C	Derivative of pGCD21; *araC*-p*ara-gfpmut1-minC*; *amy106-912;* Ap^R^ Km^R^	This work

Ap^R^, ampicillin resistance; Gm^R^, gentamicin resistance; Km^R^, kanamycin resistance; bla, betalactamase; neo, neomycin;

* *Instituto Biológico, Seção de Bacteriologia Fitopatológica, Campinas, SP, Brazil*.

For the cloning steps, we used *E. coli* DH10B (Invitrogen) cultivated in LB 0.5%-agar/LB 0.5% at 37°C (Sambrook et al., 1989). The antibiotics kanamycin, gentamicin, and ampicillin were used at the concentration of 20 μg/mL.

### Construction of the replicative plasmid expressing Xac MinC

The *minC* gene was amplified by PCR using Xac genomic DNA as a template and the primers minC_pLAL1F/minC_pLAL1R (all oligonucleotides are listed Table S1). The resultant DNA fragment was digested with *Eco*RI/*Hind*III and ligated into the Xac expression vector pLAL1 (GenBank KP696472; Lacerda et al., 2017) digested with the same enzymes, which resulted in pGCD1C.

### Vector for Xac *minC* deletion

Two DNA fragments flanking the genomic sequence of *minC*, minCup (870 bp), and minCdown (929 bp) were obtained by PCR using the primer pairs minCupF/minCupR and minCdownF/minCdownR, respectively. The PCR products minCup and minCdown were digested with the restriction enzymes *Bam*HI/*Xba*I and *Xba*I/*Hind*III, respectively, and ligated to pNPTS138 (kindly donated by Lucy Shapiro, Stanford University, USA) digested with *Bam*HI/*Hind*III, which generated pNPTSΔ*minC*.

### Vector for GFP-fusions and *gfp-minC* vector

GFP-MinC was expressed using the integrative vector pGCD21 (this work), a derivative of pHF5Ca (Ucci et al., 2014), which enables the expression of GFP (gfp-mut1, GenBank ADF80258.1) fusion proteins in Xac under the control of the arabinose promoter. To construct pGCD21, *gfp* was removed from pPM7g (Martins et al., 2010) using the restriction enzyme *Bam*HI/*Xba*I; the isolated *gfp* cassette was ligated to the backbone of pHF5Ca digested with the same enzymes, giving rise to pGCD19 (GenBank KJ619486). Next, the arabinose repressor and promoter (*araC-para*) were amplified by PCR using pEB304 (Gully et al., 2003) as a template and the primer pair pARAF/pARAR. The PCR product was digested with *Eco*RI/*Bgl*II and ligated to the backbone of pGCD19/*Eco*RI/*Bgl*II, generating pGCD21 (GenBank KU678206). In order to clone Xac *minC* in pGCD21, the gene was isolated by PCR using genomic DNA and the primers minCF/201402minCR. The PCR product was digested with *Not*I/*Xba*I prior to ligation into pGCD21/*Not*I/*Xba*I, which resulted in pGCD2C.

All the constructs were checked by DNA sequencing.

### Xac *minC* knockout

All plasmids were transformed in Xac by electrotransformation (Ferreira et al., 1995). First, Xac was transformed with the replicative vector pGDC1C, which provides an additional copy of *minC*. Afterwards, Xac/pGCD1C was transformed with pNPTSΔ*minC*, which mediates removal of chromosomal *minC* by allele exchange. Mutant strains carrying one of the first crossover events (integration of pNPTSΔ*minC* into minCup or minCdown) were selected on NYGA/kanamycin. To obtain the second crossover, deletion of *minC*, individual colonies were cultivated for 16 h in NYGB supplemented with 0.01% arabinose and gentamycin only, which is the marker of pGCD1C. The final selection of Xac/pGCD1C Δ*minC* was carried out in NYGA supplemented with 3% sucrose. Deletion of *minC* was verified by diagnostic PCR and Southern Blot experiments using *minCD* as a probe. To cure pGCD1C, Xac/pGCD1C Δ*minC* was cultivated for ~20 generations without gentamycin (Xac Δ*minC*).

Xac Δ*minC* was complemented with GFP-MinC by transformation with pGCD2C. The vector was stably integrated into the *amy* locus (Xac Δ*minC amy::gfp-minC*). For a GFP only control strain, Xac Δ*minC* was transformed with pGCD21. The vector was stably integrated into the *amy* locus (Xac Δ*minC amy::gfp*). To test whether GFP-MinC was expressed as full-length protein and not proteolytically cleaved inside the cell, cell extracts of cells expressing GFP only or GFP-MinC were analyzed by SDS-PAGE/Western Blot using antibodies against GFP (Figure S1).

### Microscopy and data analysis

Xac cells were grown to exponential phase in either NYGB or Xam1 medium and directly imaged or labeled with a fluorescent dye prior to imaging.

DAPI (4′,6-diamidino-2-phenylindole) was used to image DNA. Exponential phase cells were harvested, resuspended in 10 μM DAPI in Phosphate Buffered Saline (PBS) buffer, pH 7, for 30 min, washed twice in PBS and imaged.

HADA (Hydroxycoumarin-carboxylic acid-Amino-D-Alanine) labeling, to detect sites of peptidoglycan synthesis, was done as previously described by Kuru et al. (2015). HADA was added to 125 μM in exponential phase Xac cells, labeling time was 24 min in Xam1 or 10 min in NYGB (8% of doubling time under these growth conditions). Cells were either imaged directly when simultaneous detection of GFP was required, or fixed with ice-cold ethanol for 10 min, and washed before imaging.

Cells were imaged using a Nikon Ti-E microscope (Nikon Instruments, Tokyo, Japan) equipped with a Hamamatsu Orca Flash 4.0 camera. Image analysis was performed using the software ImageJ (http://rsb.info.nih.gov/ij/).

Xac *amy::gfp* (wild type control) and Xac Δ*minC amy::gfp* were analyzed to observe MinC deletion effects. Xac Δ*minC amy::gfp-minC* was analyzed to see if GFP-MinC could complement the Δ*minC* phenotype. GFP producing strains were used as the cytosolic GFP allowed for an easier determination of whether a cell had divided or not. First, images were manually inspected for short filamentation, minicells and branching. Two biological replicates were analyzed with at least 200 cells each. Subsequently, cell lengths of rod-shaped cells (not minicells or branched cells) were measured using the public domain program Coli-Inspector, which runs under ImageJ in combination with the plugin ObjectJ, written by Norbert Vischer (http://simon.bio.uva.nl/objectj/). After the automatic selection process, a manual check was done to make sure only single cells were selected. Cells with a length equal to or longer than 3.15 μm were defined as short filaments. Two biological replicates of each strain were analyzed with at least 500 cells per replicate.

The nucleoid length in cells of Xac *parB::parB-gfp* and Xac Δ*minC parB::parB-gfp* stained with DAPI was manually measured using ObjectJ.

Xac *amy::gfp-zapA* and Xac Δ*minC amy::gfp-zapA* were were analyzed to observe co-localization of HADA (peptidoglycan incorporation) with GFP-ZapA (division sites), and possible MinC deletion effects. After the pictures were taken, the fluorescence channels CFP (HADA) and FITC (GFP-ZapA) were merged. Linescans were made of the cells displayed using the plot profile function of ImageJ. The maximum fluorescence intensity of each cell was defined as 100%, the minimum at 0%, and plots were made relative to those levels (Figure S5). Linescans confirmed that colocalization could be scored manually from image overlays, which was done for a total of more than 100 cells per strain.

## Results

### Xac deleted for *minC* displays a branching phenotype

**Figure 1:**
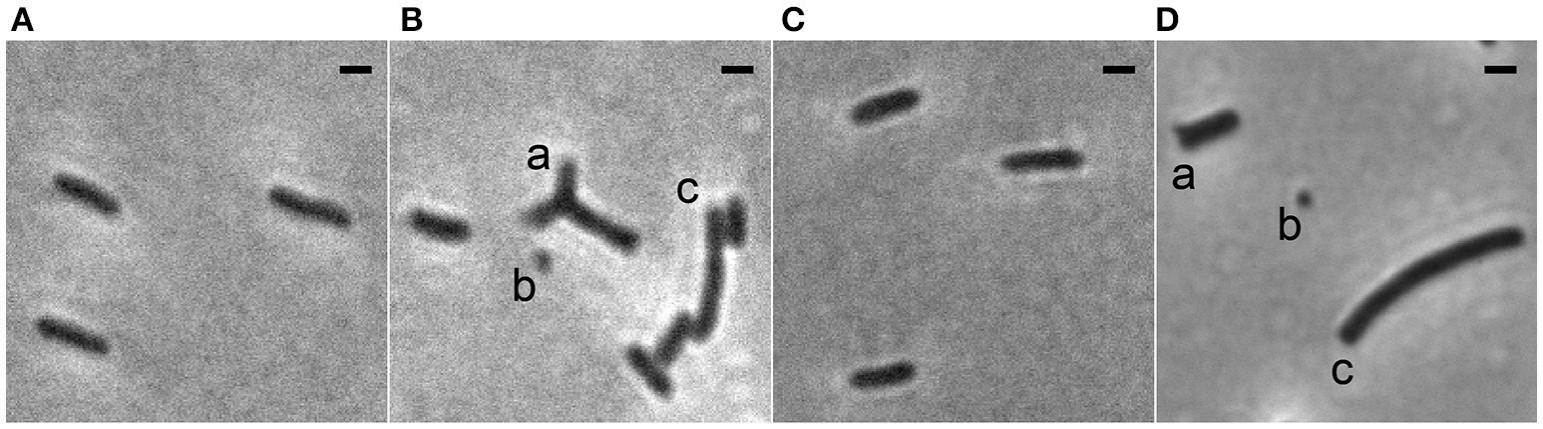
Phase contrast images showing the morphology of Xac strains grown to exponential phase in Xam1 medium. **(A)** Xac *amy::gfp*. **(B)** Xac Δ*minC amy::gfp* with (a) branching, (b) minicells, and (c) short filamentation. **(C)** Xac Δ*minC amy::gfp-minC*. **(D)** Xac Δ*minC* with (a) branching, (b) minicells, and (c) short filamentation. Scale bar: 1 μm.

To study the role of the Min system in Xac we deleted *minC* in a strain that expresses *gfp*. This resulted in a strain that exhibited the classical Δ*min* phenotype: next to rod-shaped cells of normal length, minicells and longer cells, both the result of polar divisions, could be observed (Figure 1Bb,c). However, in addition to these phenotypes, branched cells were also observed in the *minC* mutants (Figure 1Ba). Similar results were obtained in a strain that did not carry a copy of the *gfp* gene (Figure 1D). To show that the phenotype was due to the deletion of *minC*, and not caused by any polar effects, we complemented the strain with *gfp-minC* at the ectopic *amy* locus. This complementation restored the wild type phenotype (Figures 1A,C, Table 2).

**Table 2:** Phenotype observations in different strains.

**Strain**	**Minicells**	**Branching**	**Short filaments^a^**	**Normal**	**Total**
Xac *amy::gfp*	ND^b^	ND^b^	1.4 ± 0.6%	98.6 ± 0.6%	1,599
Xac Δ*minC amy::gfp*	7.3 ± 2.5%	12.9 ± 2.7%	16.0 ± 2.9%	63.8 ± 3.0%	424
Xac Δ*minC amy::gfp-minC*	ND^b^	ND^b^	1.9 ± 1.6%	98.1 ± 1.6%	1,624

a *Cells with length ≥ 3.15 μm*.

b *Not detected*.

Branches and minicells were not observed in the wild type strain nor in the strain complemented with *gfp-minC*. Branches and minicells could not be detected automatically, thus the frequency of their occurrence in the *minC* deletion strain was scored by visual inspection, which revealed that around 20% of the cells were either minicells or branched cells (Table 2, see Section Branching in the *minC* Deletion Strain is Dependent on Growth Medium for more details on branching). Subsequently, the length of the rod-shaped cells was determined by automatic length analysis using Coli-inspector (methods). This showed that the length distribution of the *minC* deletion strain was a lot broader than that of the wild type and *gfp-minC* complemented strain (Figure S2). This is most likely because the longer cells in the *minC* deletion population are the result of the outgrowth from an asymmetric division. It is impossible to precisely determine which cells are derived from an asymmetric division. Therefore, we defined “short filaments,” which are likely to represent such cells, as cells with a length of over 3.15 μm (this is 1.5 times the median length of cells in the wild type strain). These short filaments comprised around 16% of the cells observed in the *minC* deletion strain, whereas the wild type strain, and the strain complemented with *gfp-minC* had less than 3% of these cells. As the *gfp-minC* complemented strain is so similar to the wild type, while the *minC* knockout is strikingly different, and as GFP-MinC does not show signs of degradation even when overexpressed (Figure S1), we conclude that GFP-MinC is fully functional.

### MinC is localized at the poles and oscillates from pole to pole

**Figure 2:**
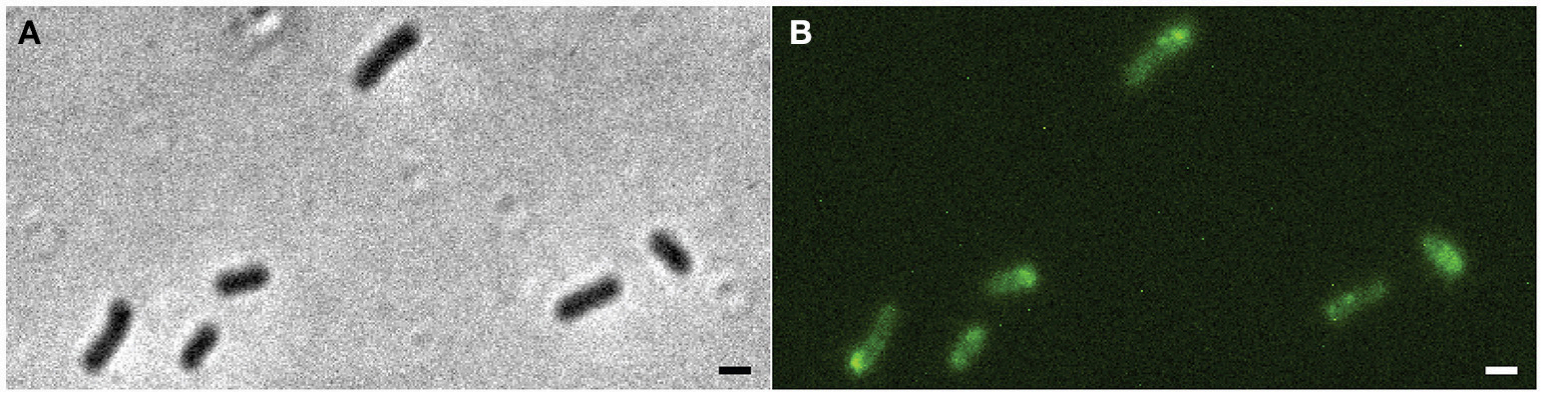
Xac Δ*minC amy::gfp-minC* grown to exponential phase in Xam1 medium. **(A)** Left: phase contrast showing phenotype similar to wild-type. Right: FITC showing GFP-MinC located mostly at cell poles. **(B)** Line scans showing fluorescence intensity over a line drawn along the central axis of cells numbered in the picture. Scale bar 1 μm.

**Figure 3:**
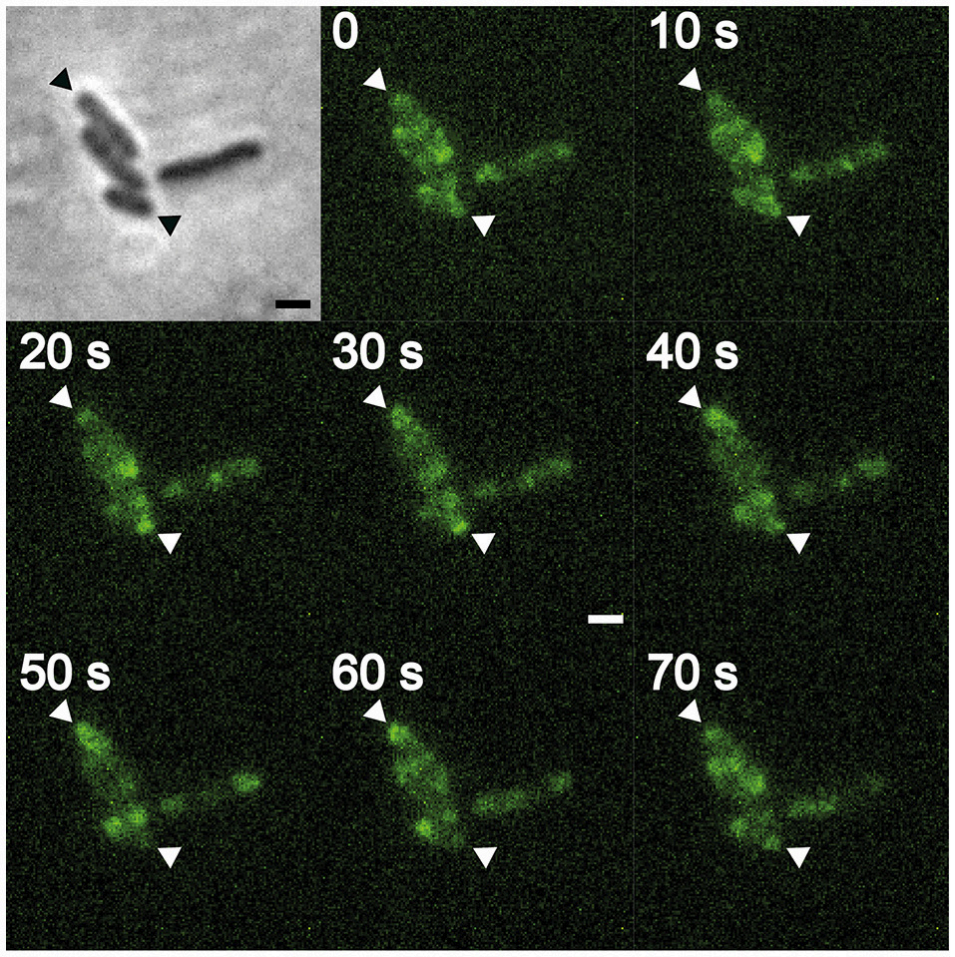
Xac Δ*minC amy::gfp-minC*, contrast (inset), and FITC time lapse. Triangles indicate cell poles showing MinC oscillation pattern. Scale bar 1 μm.

The strain Xac Δ*minC amy::gfp-minC*, in which *gfp-minC* is the only copy of *minC*, was studied using fluorescence microscopy. As expected, GFP-MinC was localized in a gradient throughout the cell with the maximum at one of the poles (Figure 2, Figure S4). To see whether MinC oscillates from pole to pole, as previously described for *E. coli* and recently *S. elongatus* (MacCready et al., 2016), we imaged the cells with 10 s intervals. These short time lapses clearly show that GFP-MinC localization is dynamic, and that the protein oscillates from pole to pole (Figure 3, Movie S1). Following several oscillations in various cells over time revealed that GFP-MinC oscillates with a periodicity of roughly 65 s, which is comparable to the oscillations described in *E. coli* (Raskin and De Boer, 1999a).

### Branching in the *minC* deletion strain is dependent on growth medium

**Figure 4:**
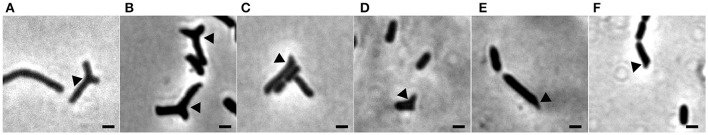
Phase contrast images showing branching of Xac Δ*minC amy::gfp* grown to exponential phase in different media. **(A)** Xam1. **(B)** Xamg1. **(C)** LB 0%. **(D)** LB 0.5%. **(E)** NYCB. **(F)** NYGB. Scale bar 1 μm.

The branching phenotype of *E. coli min* mutants has been described in two studies from the 1990's (Akerlund et al., 1993; Gullbrand et al., 1999), but the phenotype, to paraphrase a paper from the Young lab, has received much less attention than other questions related to bacterial morphology (Potluri et al., 2012). Branching of *min* mutants was shown to be dependent on the composition of the growth medium. Some branching was observed on rich (LB) medium, whereas growth on minimal salt (M9) media supplemented with casaminoacids and either succinate or acetate, but not glucose, resulted in branching in 5–20% of the cells depending on the *E. coli* strain studied (Akerlund et al., 1993; Gullbrand et al., 1999). We tested the medium dependency of the branching phenotype of the Xac *minC* deletion strain and observed similar results: hardly any branches on the rich NYG/CB media and roughly 12% of branches on the minimal salt Xam1 medium. An intermediate phenotype was observed on LB where cells showed bulges and abnormal extensions at the poles. These cells were scored as branching cells (Table 3; Figure 4). We would like to remark that it is formally possible that minicells do not only originate from polar divisions but also from budding of branches. However, as the frequency of minicelling under non-branching conditions is almost the same as on branching conditions we deem this unlikely.

**Table 3:** Xac Δ*minC amy::gfp* phenotypes in different media.

**Medium**	**Minicells**	**Branching**	**Short Filaments**	**Normal**	**Total**
Xam1	7.3 ± 2.5%	12.9 ± 2.7%	16.0 ± 2.9%	63.8 ± 3.0%	424
Xamg1	16.3 ± 4.0%	18.0 ± 5.6%	13.4 ± 4.4%	52.4 ± 6.1%	401
LB 0%	11.6 ± 1.6%	6.9 ± 0.4%	18.8 ± 0.6%	62.8 ± 4.2%	262
LB 0.5%	11.6 ± 1.3%	8.7 ± 3.4%	17.7 ± 1.7%	62.0 ± 9.5%	387
NYCB	12.4 ± 2.2%	0.78 ± 0.03%	17.4 ± 2.2%	69.5 ± 2.3%	387
NYGB	11.7 ± 3.4%	1.4 ± 1.9%	15.3 ± 3.5%	71.6 ± 1.2%	488

### Nucleoid organization in the Xac *minC* deletion strain

**Figure 5:**
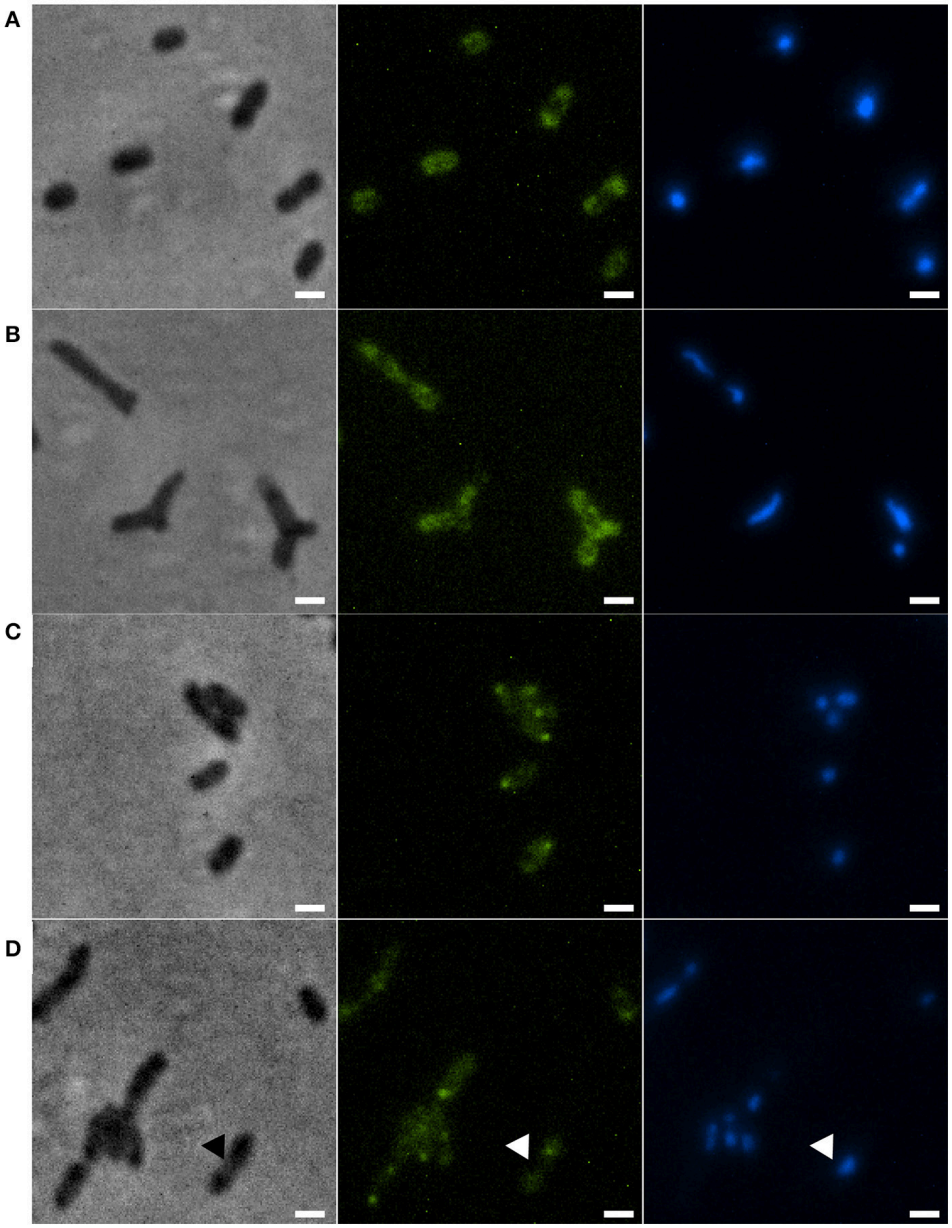
**(A)** Xac *parB::parB-gfp* grown in Xam1. **(B)** Xac Δ*minC parB::parB-gfp* grown in Xam1. **(C)** Xac *parB::parB-gfp* grown in NYGB. **(D)** Xac Δ*minC parB::parB-gfp* grown in NYGB, triangles indicate divisions initiated over non-segregated nucleoids. All cells in the figure were labeled with DAPI. Phase contrast (left), DAPI (center) exhibiting nucleoids, and FITC (right) exhibiting ParB-GFP. Scale bar: 1 μm.

**Figure 6:**
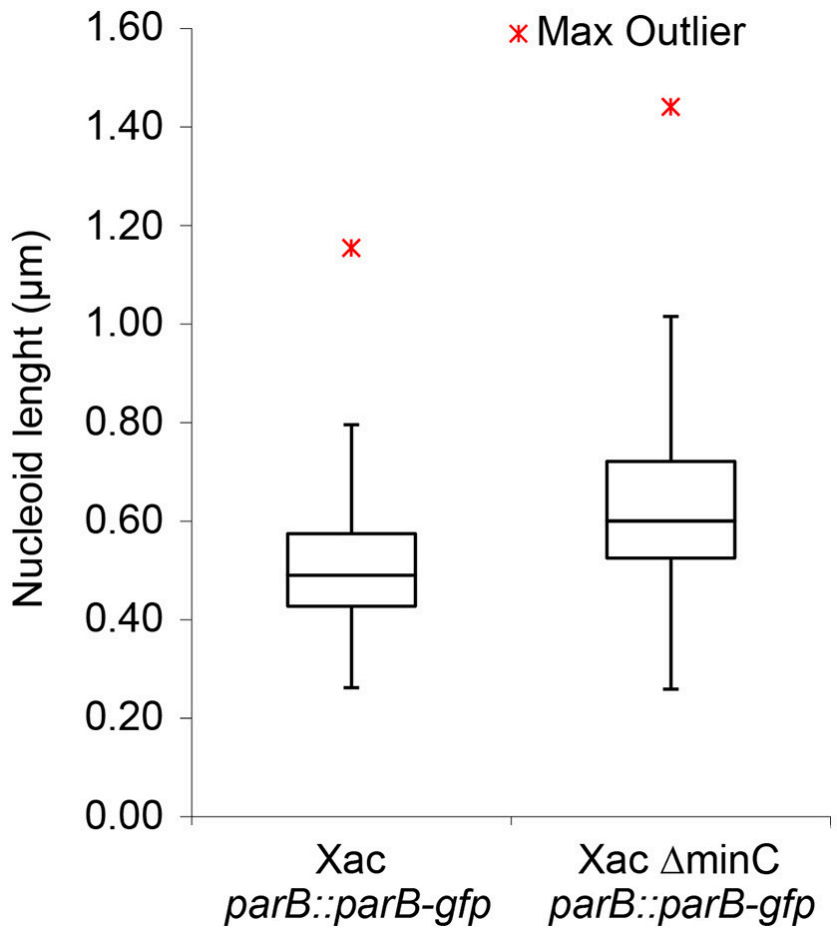
Nucleoid length of Xac cells, with and without MinC, grown to exponential phase in Xam1 medium, measured manually with ObjectJ. Whiskers at the top represent the 1.5 interquartile range and whiskers at the bottom extend to the minimum value. *p* < 0.001 (Mann-Whitney *U*-Test).

Disruption of the *min* system in *E. coli* (Jaffé et al., 1988; Mulder et al., 1990) results in aberrant nucleoids, and in the case of branching cells large masses of DNA are often located at branching points (Akerlund et al., 1993). To study nucleoid distribution and organization we made use of strains carrying a functional GFP fusion to ParB (Ucci et al., 2014). In a wildtype background, GFP-ParB localizes to the origin-proximal *parS* site on the chromosome. Upon *parS* duplication during chromosome replication and segregation, ParB bound to the second *parS* copy moves from one pole to the other during the cell cycle (Ucci et al., 2014; Figure 5A). We introduced the *parB-gfp* allele into the Δ*minC* background to construct the strain Xac Δ*minC parB::parB-gfp*, and studied nucleoid distribution with DAPI and nucleoid organization using ParB-GFP. In branching cells, we observed longer nucleoid length and the occasional accumulation of DNA at branch points (Figures 5B, 6), although the accumulation at branch points was not a typical feature of branching cells (9 out of 36 branching cells on Xam1 had DNA at the branch point). The ParB-GFP pattern appeared less clearly defined on Xam1 medium (Figures 5A,B) with more faint fluorescent spots visible in the cells compared to the one or two spots per cell seen with NYGB medium (Figures 5C,D).

In non-branching Δ*minC* cells the organization of the nucleoid looked more like wild type—the DNA was clearly more condensed—although in some cells it appeared that divisions were initiated over non-segregated nucleoids (Figure 5D). Combined, these results suggest that chromosome segregation/organization is affected in the absence of MinC.

### Branching cells have deficiencies in positioning cell division and peptidoglycan synthesis

**Figure 7:**
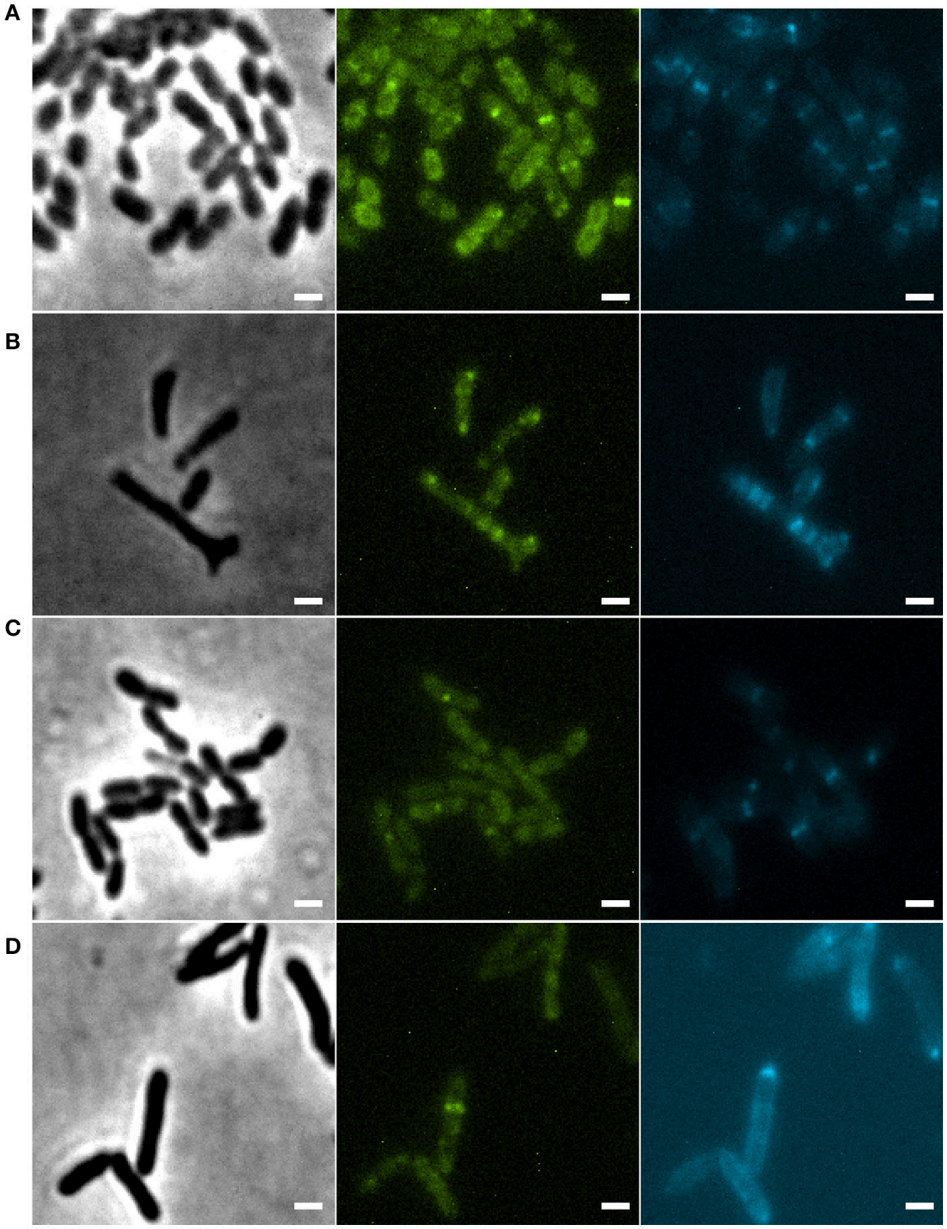
**(A)** Xac *amy::gfp-zapA* grown to exponential phase in Xam1, labeled with 125 μM of HADA for 24 min. **(B)** Xac Δ*minC amy::gfp-zapA* grown to exponential phase in Xam1, labeled with 125 μM of HADA for 24 min. **(C)** Xac *amy::gfp-zapA* grown to exponential phase in NYGB, labeled with 125 μM of HADA for 10 min. **(D)** Xac Δ*minC amy::gfp-zapA* grown to exponential phase in NYGB, labeled with 125 μM of HADA for 10 min. Phase contrast (left). FITC (center) exhibiting GFP-ZapA at the septum. CFP (right) exhibiting HADA (peptidoglycan incorporation sites) mostly at the septum. Scale bar 1 μm.

In *E. coli*, branch formation is the result of defects in positioning the cell division machinery, notably FtsZ (Potluri et al., 2012). In *min* mutants the FtsZ ring can form at the poles, but there is also an increased zone for FtsZ polymerization at midcell, which also occurs in cells with defective PBP5 (Potluri et al., 2012). In *E. coli* some peptidoglycan (PG) is synthesized in an FtsZ-dependent manner at a site that will form the new pole, and strikingly this PG is not subject to turnover of material, which is why it is called inert PG (iPG) (de Pedro et al., 1997). iPG is always found at the poles and at the tips of branches, which originate from places on the side wall that contained iPG. We studied the relation between cell division site placement and peptidoglycan synthesis by using GFP-ZapA (Martins et al., 2010) as a proxy for division site localization and the fluorescent D-amino acid analog HADA (Kuru et al., 2012) to label sites of active, ongoing PG synthesis. Cells were grown for 24 min (8% of the doubling time) in the presence of HADA and analyzed. In wildtype cells, most PG synthesis occurred at division sites at midcell, which could also be identified by fluorescent bands of GFP-ZapA (Figures 7A,C). This pattern is similar to HADA labeling in other rod-shaped bacteria such as *E. coli* and *B. subtilis* (Kuru et al., 2012). Sometimes, spots of HADA were seen at some poles, which could be due to the fact that the HADA was incorporated in the septum just before cell division. In non-dividing cells, GFP-ZapA localized throughout the cytosol as described (Martins et al., 2010). Consistent with strong PG incorporation at division septa, 97% of the cells that had clear labeling with both HADA and GFP-ZapA showed an overlapping signal (Table 4). In the *minC* deletion mutant, this colocalization of GFP-ZapA and HADA incorporation at division sites was lost (Figures 7B,D, Table 4). The *minC* deletion strain when grown in conditions that promote branching, shows less complete GFP-ZapA rings, which also do not appear to be fully perpendicular to the cell axis, and strong GFP-ZapA signals are not overlapping with bands of HADA that can be observed at sites that show constrictions that would be indicative of ongoing divisions. When these cells are grown under non-branching conditions, this defect in colocalization of GFP-ZapA and HADA was not rescued (Table 4). Recent work has pointed at a role for metabolism in the control of cell division, with pyruvate levels in *B. subtilis* stimulating efficient Z ring formation under nutrient rich conditions through pyruvate dehydrogenase E1α (Monahan et al., 2014; Sperber and Herman, 2017). We tested whether the addition of pyruvate to the medium in which branching occurs can rescue Z ring positioning and branching, but this was not the case (Figure S3).

**Table 4:** Overlapping events in pictures of cells with HADA and GFP-ZapA signals.

**Strain**	**Medium**	**Overlap**	**No overlap**	**Total**
Xac *amy::gfp-zapA*	Xam1	97% (101)	3% (3)	100% (104)
Xac Δ*minC amy::gfp-zapA*	Xam1	18% (25)	82% (114)	100% (139)
Xac *amy::gfp-zapA*	NYGB	73% (76)	27% (28)	100% (104)
Xac Δ*minC amy::gfp-zapA*	NYGB	16% (19)	84% (102)	100% (121)

## Discussion

In this work, we have shown that MinC oscillates from pole to pole to ensure proper cell division and cell shape in Xac. Although the oscillation of MinCD was described nearly 20 years ago (Raskin and De Boer, 1999a,b), it was only last year that the second and third cases of oscillating Min proteins in the homologous host were described in *S. elongatus* (MacCready et al., 2016) and *V. cholerae* (Galli et al., 2016). Our observation of GFP-MinC oscillations in Xac now provides the fourth example. Oscillations of Min system proteins have also been reported in heterologous systems. For example, MinDE from *Neisseria gonorrhoeae* have been shown to oscillate when expressed in *E. coli* (Ramirez-Arcos et al., 2002), and MinDE from *Clostridium difficile* have been shown to oscillate when expressed in *B. subtilis* (Makroczyová et al., 2016). Combined, these reports support the idea that an oscillating Min system is a common feature as long as the Min system protein MinE is present.

The Min system is responsible for ensuring that the cell division machinery assembles at the right place; this goal is achieved by negative regulation of FtsZ by MinC (Rowlett and Margolin, 2015). The absence of MinC creates more space for Z rings to assemble, both at the poles of cells and at midcell, resulting in the formation of minicells, the characteristic phenotype of *min* mutants. The presence of other regulatory systems, such as nucleoid occlusion and the composition of the cell wall, generally confine Z rings at midcell sufficiently to ensure “normal” divisions, perpendicular to the length of the cell, but under certain conditions branches can start to form. Branching is thought to arise from a disruption in the organization of Z rings in such a way that Z rings are allowed to form in more places and at unnatural angles. This is in line with the observation that branching in *E. coli* is exacerbated upon overexpression of *ftsZ* (Potluri et al., 2012), as higher levels of FtsZ result in the formation of abnormal rings in multiple bacteria. Branching of *min* mutants of *E. coli* does not always occur—but seems to be dependent on growth conditions and correlated with the general physiology of the cell rather than specific media components (Gullbrand et al., 1999). We have observed a similar, growth condition dependent, branching phenotype in Xac.

Several papers in the past decade have pointed to a role for metabolism in the control of cell division and shape (Sperber and Herman, 2017). For example, Z ring formation is directly controlled in *E. coli* and *B. subtilis* by two different metabolic enzymes that moonlight as FtsZ inhibitors (Hill et al., 2013). *In vitro* evidence points to UDP-glucose as the molecule responsible for the metabolic control but an (additional) role for increased levels of peptidoglycan precursors in triggering enhanced division is possible (Sperber and Herman, 2017). In addition, pyruvate dehydrogenase E1α (PDH E1α) has been identified as a positive regulator of FtsZ ring formation in *B. subtilis*, which links the presence of high levels of pyruvate to increased division at midcell (Monahan et al., 2014). High levels of pyruvate are linked to a high glycolytic flux and a switch to gluconeogenic conditions has implications for the availability of cell wall precursors and FtsZ positioning (Monahan et al., 2014). In this respect it is interesting to note that the general physiology in which branching occurs most in the *min* mutants in both *E. coli* (Gullbrand et al., 1999) and Xac is gluconeogenic growth.

A branching phenotype is a common feature among asymmetric polar growing bacteria (Wells and Margolin, 2012), such as in Actinomycetes, Rhizobiales, and Caulobacterales (Brown et al., 2012). Here, branching is caused by dedicated proteins, not aberrantly positioned Z rings (Howell and Brown, 2016). In *E. coli*, positioning of FtsZ not exactly at midcell can result in the synthesis of inert PG that will form the future tip of a branch. We studied the relation between cell division and PG synthesis in the branching *min* mutants of Xac. We used the fluorescent D-amino acid analog HADA, which can be used to track PG synthesis in many different bacteria (Kuru et al., 2012), to establish that wild type Xac grows similar to other rod-shaped bacteria, with PG incorporated both at the lateral wall and at division sites. In wild type cells, PG synthesis at division sites and visible GFP-ZapA rings clearly overlapped. In the branching *minC* mutant, these patterns were lost. Cell division was clearly impaired as shown by the scattered localization of ZapA patches that often did not form perpendicular rings that would support division, and scattered HADA incorporation. Interestingly, the ZapA and HADA localization patterns hardly overlapped in the *minC* mutant, something one would expect if the aberrant placement of division sites would recruit PG synthesis enzymes.

Potluri et al. (2012) observed similar branching formation in *E. coli* cells deleted for PBP5 and other low molecular weight penicillin-binding proteins (PBPs). They proposed a link between branching formation and aberrant cell division. In the absence of PBPs, and PBP5 in particular, the organization of Z rings is disturbed in such a way that they are allowed to form in more places and at unnatural angles. This leads to malformation of daughter cells, which leads to branching phenotypes. Our observations of GFP-ZapA and HADA incorporation demonstrate that MinC knockout in Xac leads to disruption of both cell division and peptidoglycan incorporation, but the effects on Z-ring placement seem more drastic compared to the *E. coli* phenotype. However, it is likely that the disruption of division site placement and peptidoglycan incorporation result in similar branching of daughter cells as previously reported for *E. coli* (Potluri et al., 2012).

Finally, we observed that similar to *E. coli min* mutant branching cells, Xac *min* mutants that branch, show disorganization of the nucleoids. Whether this is a result of the deformation of the cells only, or whether the *min* system influences asymmetric chromosome segregation in Xac we cannot say. We noticed that ParB-GFP localized in clear spots in cells grown on NYGB medium, whereas the localization pattern was more faint in Xam1 medium. We don't know the cause of this difference - but it is probably not caused by the effect of the media on GFP as GFP-ZapA did not show such a difference.

In conclusion, we have shown that Xac *min* mutants display a metabolism-dependent branching phenotype, which is the result of delocalized cell division and peptidoglycan synthesis. Also, we have shown that Min oscillation occurs in a fourth organism next to *E. coli, S. elongatus*, and *V. cholerae*. Although our study focused on Xac cell biology, the described GFP-fusions and HADA-labeling will be used as tools to characterize the mode of action of potential antibacterials against the plant pathogen Xac (see Silva et al., 2013; Król et al., 2015). Characterizing such antibacterials is ongoing in our laboratories.

## Author contributions

HF and DS conceived the study. AL, GD, HF, and DS designed the research. GD constructed the plasmids and strains. AL and TB performed the microscopy experiments. AL quantified the morphological data and assembled the pictures and graphs. AL, GD, HF, and DS analyzed the data. AL, HF, and DS wrote the manuscript. All authors have read and approved the final manuscript.

### Conflict of interest statement

The authors declare that the research was conducted in the absence of any commercial or financial relationships that could be construed as a potential conflict of interest.
