# Supplementary material for: Xanthomonas citri MinC Oscillates from Pole to Pole to Ensure Proper Cell Division and Shape
Source: Front Microbiol. 2017 Jul 19;8:1352. doi: 10.3389/fmicb.2017.01352 (PMC5515816; doi:10.3389/fmicb.2017.01352)
Supplement: Supplementary file 1 [file Image1.PDF]

Supporting information for:

Xanthomonas citri MinC oscillates from pole to pole to ensure proper cell division and shape

André Soibelmann Glock Lorenzoni[a], Giordanni Cabral Dantas[b], Tessa Bergsma[a], Henrique Ferreira[b], Dirk-Jan Scheffers[a,*].

Movie S1. GFP-MinC oscillations - 15x sped up timelapse of images shown in Fig. 3, in total 10 frames taken over 50 sec, looped 9 times.

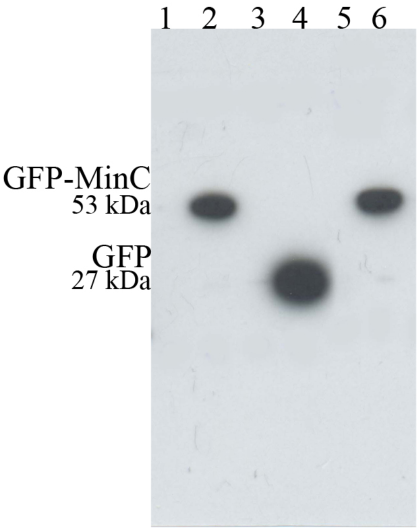

Figure S1. Western blot with cells grown in Xam1 with 2 % glucose using Rabbit polyclonal GFP[1] antibody. Secondary antibody detection was done using the Amersham ECL kit.

1 Xac *amy::gfp-minC*
2 Xac *amy::gfp-minC* + arabinose[2]
3 Xac *ΔminC amy::gfp*
4 Xac *ΔminC amy::gfp* + arabinose[2]
5 Xac *ΔminC amy::gfp-minC*
6 Xac *ΔminC amy::gfp-minC* + arabinose[2]

[1]G1544 from SIGMA diluted 1:2500.
[2]0.05 % arabinose during 1 h.

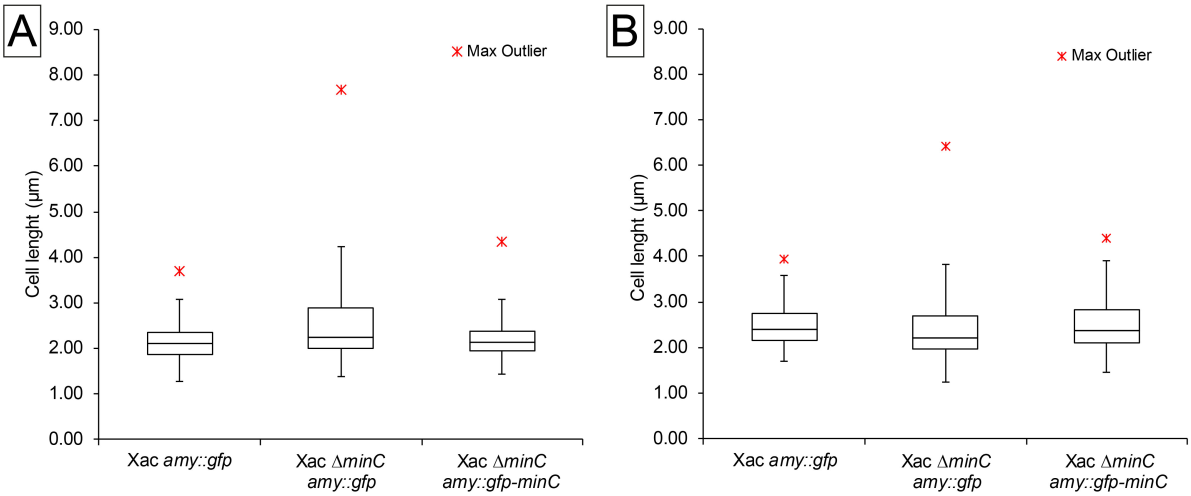

Figure S2. A) Cell length of Xac *amy::gfp* (n = 1599), Xac Δ*minC amy::gfp* (n = 1114) and Xac Δ*minC amy::gfp-minC* (n = 1624), grown to exponential phase in Xam1 medium. **B)** Cell length of Xac *amy::gfp* (n = 729), Xac Δ*minC amy::gfp* (n = 589) and Xac Δ*minC amy::gfp-minC* (n = 551), grown to exponential phase in NYGB medium. Both sets were measured automatically with Coli-Inspector, plugin for ObjectJ. Whiskers at the top represent the 1.5 interquartile range and whiskers at the bottom extend to the minimum value.

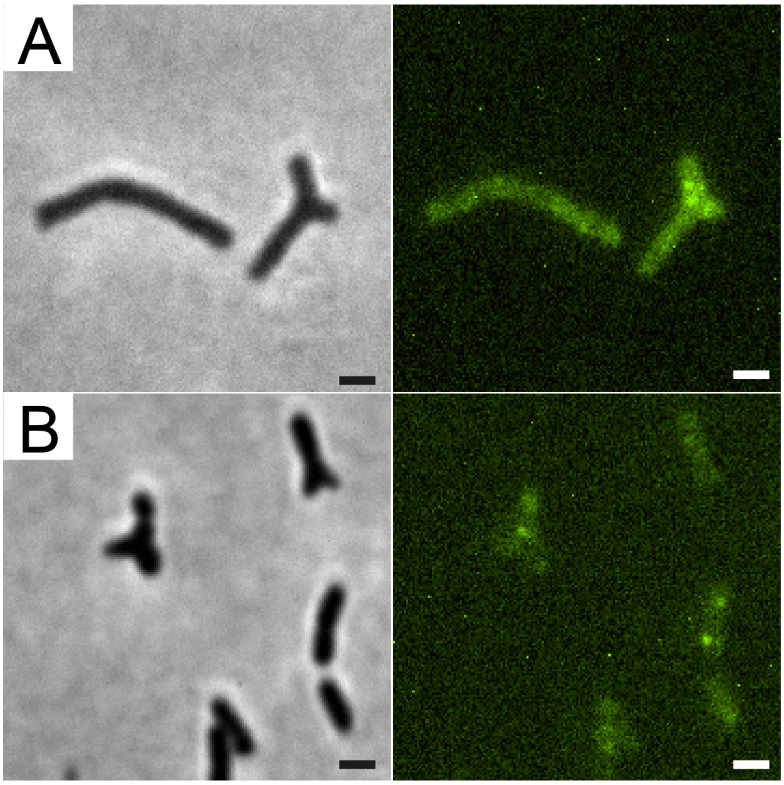

Figure S1. Xac Δ*minC amy::gfp* grown to exponential phase in NYGB medium diluted to Xam1 medium and then grown overnight. **A)** without sodium pyruvate . **B)** with 1 % sodium pyruvate. Scale bar: 1 μM.

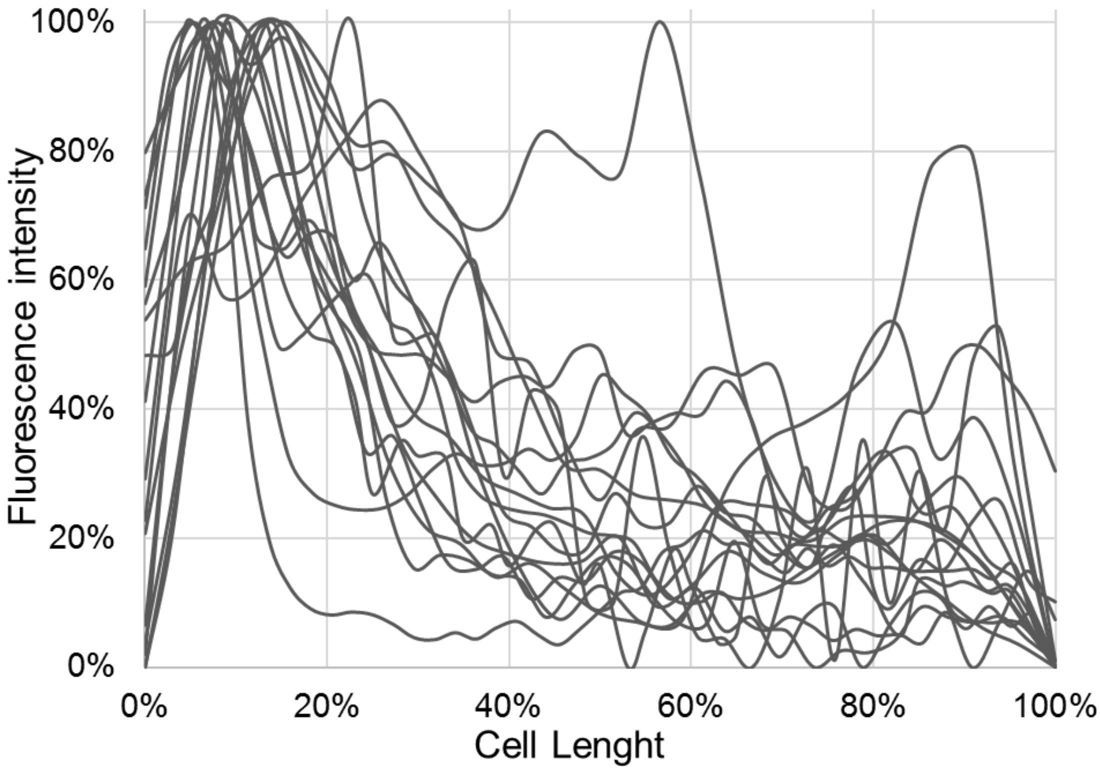

Figure S2. Line scans showing relative fluorescence intensity of FITC channel (GFP-MinC) along the central axis of Xac Δ*minC amy::gfp-minC* cells grown to exponential phase in Xam1 medium.

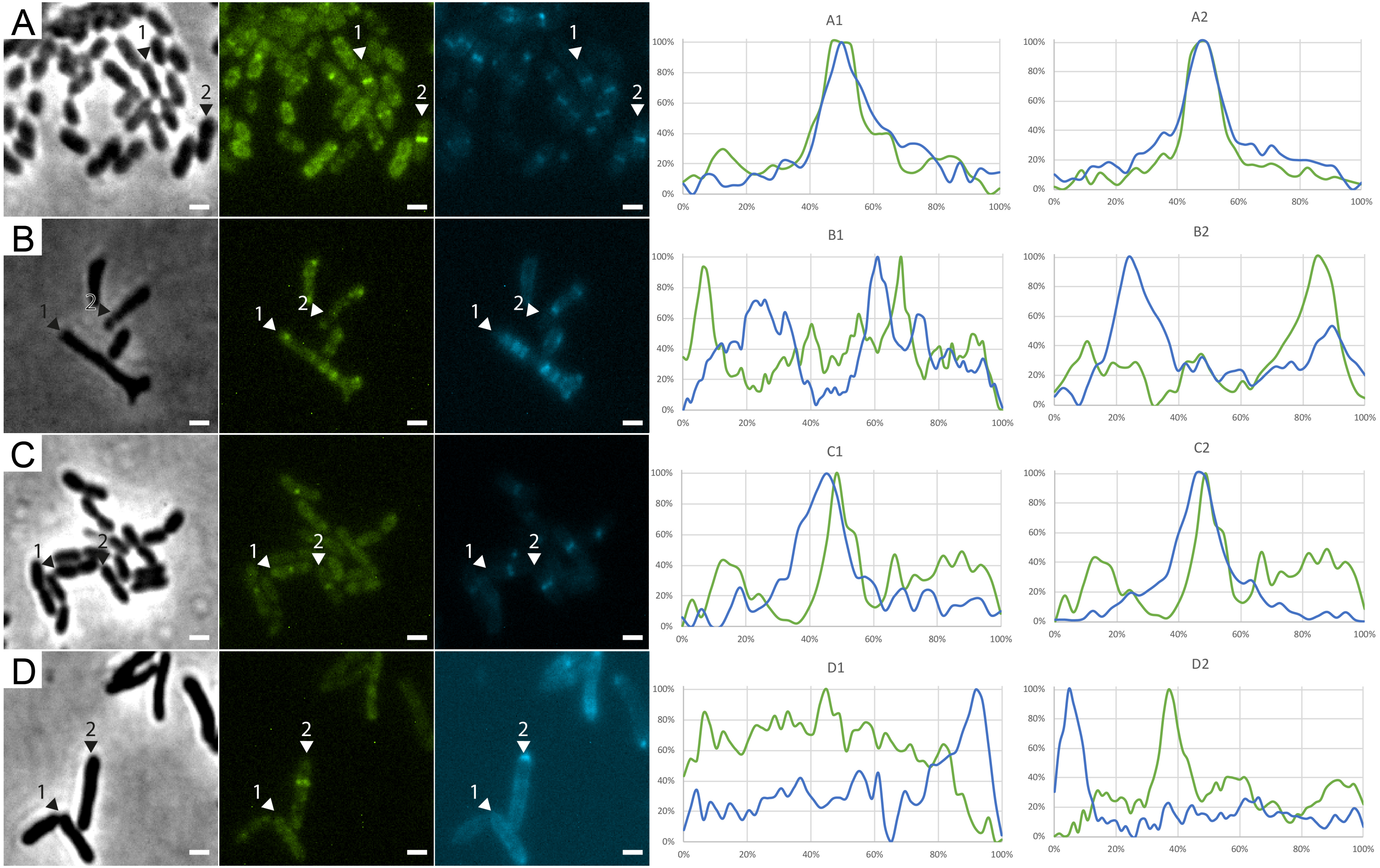

Figure S3. Same as **Figure 7**, and including line scans of respective cells on the left side, lines in green are fluorescence intensity of FITC (GFP-ZapA) whereas lines in blue are fluorescence intensity of CFP (HADA).

Table S1. Oligonucleotides used in this study. Restriction sites underlined.

Name	Sequence
pARAF	5´- AAAGAATTCGCATAATGTGCCTGTCAAATG
pARAR	5´- TTTAGATCTTTCCTCCTGCTAGCCCAAAAAAACG
minCF	5´- TAAGCGGCCGCGTGGCAAGTGTGAATGTGGATTTTG
201402minCR	5´- AAATCTAGATCAATCAAGCGCAGCGATCTTG
minC_pLAL1F	5’- AAAGAATTCACCATGGCAAGTGTGAATGTGGATTTTG
minC_pLAL1R	5’- AAAAAGCTTTCAATCAAGCGCAGCGATCTTG
minCupF	5’- AAAGGATCCGTATGACTGAGGTATCCCAACATGTC
minCupR	5’- AAATCTAGATGCCACAACTCAGCTCCCCGTCGATG
minCdownF	5’- AAATCTAGAGATTGACGCGGCCCAACCACAGAAATATTC
minCdownR	5’- AAAAAGCTTCAATGATGATCTGCAGGCGGTTCTTGG
minCupF (*Bam*HI)	5’- AAAGGATCCGTATGACTGAGGTATCCCAACATGTC
minCupR (*Xba*I)	5’- AAATCTAGATGCCACAACTCAGCTCCCCGTCGATG
minCdownF (*Xba*I)	5’- AAATCTAGAGATTGACGCGGCCCAACCACAGAAATATTC
minCdownR (*Hind*III)	5’- AAAAAGCTTCAATGATGATCTGCAGGCGGTTCTTGG
